# Water Sources Quality in Urban Slum Settlement along the Contaminated River Basin in Indonesia: Application of Quantitative Microbial Risk Assessment

**DOI:** 10.1155/2018/3806537

**Published:** 2018-09-18

**Authors:** Sri Y. Irda Sari, Deni K. Sunjaya, Hana Shimizu-Furusawa, Chiho Watanabe, Ardini S. Raksanagara

**Affiliations:** ^1^Department of Public Health, Faculty of Medicine, Padjadjaran University, Bandung, Indonesia; ^2^Department of Human Ecology, School of International Health, Graduate School of Medicine, The University of Tokyo, Tokyo, Japan

## Abstract

Fecal contamination in water sources is still found globally, especially in urban slum areas of mid-low income countries. Fecal contamination as an indicator of low levels of hygiene and sanitation practices as well as poor management of drinking water supply might increase the risk of waterborne diseases in developing countries like Indonesia. This study aimed to assess quality of all water sources in one of the urban slum settlements along a contaminated river basin in Indonesia. A cross-sectional survey was conducted during the period of August to October 2015. Water samples were taken with simple random sampling from households in urban slum areas along the Cikapundung river basin in the center of Bandung city, Indonesia. Water samples (*n*=379) from 199 households and 15 common wells were tested for microbiological contamination, and 61 samples of ground water sources and river were tested for selected heavy metal contamination. Annual risk of infection from all water sources was calculated using the quantitative microbial risk assessment. Tap water distribution was poor in this slum area. Most of the dug wells and half of refill bottled water were contaminated. Estimated highest annual risks of infection due to fecal contamination would be caused by dug well and spring water since majority of the households did not use septic tank and disposed human waste directly to the river. Improvement in point-of-use water treatment and storage is essential to prevent risk of waterborne diseases, and tap water should be more accessible and affordable in urban slum areas. The integrated monitoring system to control the quality of refill bottled water production is one of the many essential issues to be prioritized.

## 1. Introduction

Water, sanitation, and hygiene (WaSH) are prerequisite to human health and development, since clean water is essential for human's daily living especially for drinking water. Millions of people in developing countries still have no access to adequate and safe water supply. The number of people without access to safe water in urban areas increases sharply in developing countries as a result of rapid urbanization, much of which occurred in peri-urban and slum areas since the last decade [1]. The United Nations has projected a rapid population growth in urban areas between 2000 and 2030, suggesting that 6 out of 10 people will be living in cities. Therefore, accessible and adequate safe drinking water and sanitation in urban areas particularly for urban poor dwellers should be prioritized by policy makers to decrease the risk of water-related diseases [2].

Fecal contamination in water sources, an indicator for poor sanitation and hygiene, is still found around the world, especially in slum areas of mid-low income countries [3]. Diarrhea or gastroenteritis is among the main causes of health problems; 1.5 million deaths were caused by diarrhea in 2012 and 280,000 were caused by poor sanitation [4]. In Indonesia, diarrhea is common among infants and children. Contact and ingestion of unsafe water, lack of water supply, poor personal and domestic hygiene, and inadequate development and management of water resources/water systems are known as risk factors of diarrhea in Indonesia [5].

Bandung city, a densely populated area, is the capital city of West Java Province. The local water company that is responsible for tap water distribution in Bandung city only supplies 46.91% of the public need for clean water; the remaining 53% have to find other safe water sources on their own. However, the quality of tap water from the local water company was poor according to the report from the health office that revealed only 13.33% from 210 samples had good quality. The lack of local water supply service has forced the lower class to find alternative water sources, which are usually underground water consisting mostly of borehole/wells with mechanized pumping and dug wells. These conditions will surely increase safe water demand in Bandung city [6].

Taman Sari subdistrict is one of the slum areas in the center of Bandung city with poor coverage for safe water, high-reported diarrhea cases, and high population density. This slum area is part of the priority for a development project targeting urban slum areas in 5 large cities in Indonesia. Taman Sari subdistrict is located right on the Cikapundung river basin. Cikapundung River is the main river among 15 rivers which cross the city and empties into Citarum River. Cikapundung River has been heavily contaminated by household waste and latrine disposal from settlement along the riverbanks while Citarum River, in the south part of the city, contains industrial waste contamination.

Surveillance of water source quality, especially microbiology parameters, is required to prevent further contamination. Quality is normally assessed against both microbial indicators and chemical parameters, although the microbial quality is the most important aspect from public health perspective because epidemic outbreaks caused by some pathogenic organisms can be very fast [7]. WHO guidelines for drinking water quality recommend ensuring the safety of drinking water supplies by development and implementation of risk management strategies to control hazardous constituents in water [8]. This study aimed to assess the quality of various water sources used by urban slum dwellers along one of the contaminated river basins to inform key players in the water sector to improve water quality in regards to public health protection.

## 2. Materials and Methods

This study was an observational research with cross-sectional design, which was conducted from August to October 2015. The study population was all the households in all RW (*Rukun Warga*; community association) along the Cikapundung river basin in Taman Sari subdistrict. Samples were selected by simple random sampling from the list of total households in that area (*n*=1734). Minimal samples were calculated using sample size estimations for a single proportion with alpha 5% and power 80%. Informed consent was done prior to sample collection and ethical approval was obtained from the Ethical Committee in the Faculty of Medicine, Padjadjaran University.

In all selected households, all type of water sources were collected including raw and drinking water sources. Water samples from the field were collected using sterile plastic and then stored inside a cooler box before being transported to the laboratory, afterward laboratory processing was done within 6–8 hours after retrieval. Water samples from ground water and tap water were 10x diluted (or 100x if the water was visibly cloudy) using sterile physiological saline solution. Bacterial identification was conducted by a membrane filtration technique, using the nitrocellulose membrane filter (47 mm in diameter, pore size: 0.45 *μ*m Merck Millipore) and Chromocult *Coliform* agar as culture media (Chromocult *Coliform* agar from Merck®). One hundred milliliters of the diluted sample was then filtered through membranes and placed on media. Water samples from drinking water such as bottled water and refill bottled water were not diluted; 100 ml of the water sample was directly filtered through the membrane. After that, culture media was incubated at 37°C for 24 hours until colonies formed. The sample was reported *E. coli*-positive if a purple-bluish-coloured colony was found. *Coliform* bacteria-containing samples were reported if a red-coloured-colony was found. *E. coli* and *Coliform* bacteria were identified by colony counting expressed in colony-forming units (CFU) per 100 ml water sample. Raw water was determined as contaminated if the sample shows >50 CFU/100ml based on the Indonesian guideline for clean water, and drinking water should contain 0 CFU/100 ml *Coliform* and *E. Coli* in the sample.

Identification of microbiology parameters was conducted in the Laboratory of Microbiology and Parasitology, Faculty of Medicine, Padjadjaran University. Sixty-one water samples from ground water sources and rivers were selected to be tested for heavy metal contamination. This chemical examination was conducted using ICP-MS (Inductively Coupled Plasma Mass Spectrometry) which has greater speed, precision, and sensitivity compared to atomic absorption spectroscopy. ICP-MS is capable to detect metals and several nonmetals at concentrations as low as one part in 10^15^ (part per quadrillion, ppq) on noninterfered low-background isotopes by ionizing the sample with inductively coupled plasma and then using a mass spectrometer to separate and quantify those ions. The validation was prepared by the certified reference material “NIST 1643e Trace Element in Water” using Agilent 7500ce ICP-MS. This test was done in the Department of Human Ecology, School of International Health, the University of Tokyo, Japan.

The data were further analysed using the quantitative microbial risk assessment (QMRA) approach in order to identify the risk of infection from various water sources by focusing on the microbiological quality of water sources. Equations that were used in this study are as follows:

(1) $$\begin{array}{c} d=V\times c, \end{array}$$

(2) $$\begin{array}{c} P_{\text{I}}=1-{\left[1+\left(\frac{d}{N_{50}}\right)\right]}^{-\alpha }, \end{array}$$

(3) $$\begin{array}{c} P_{\text{annual}}=1-{\left(1-P_{\text{infection}}\right)}^{365}. \end{array}$$

In Equation (1), *d* is ingested dose per day, *V* is volume of water consumed per day, and *c* is concentration of microorganism in water. The volume of water consumed per day is defined as 1.1 liter per person as derived from the previous study [9]. In Equation (2), *P*_I_ is the probability of infections to occur, *α* is the parametric slope, and *N*_50_ is the average dose to cause an infection (*E. coli*: *N*_50_ = 8.6 × 10^7^, *α* = 0.1778). In Equation (3), *P*_annual_ is the probability of annual infection, and *P*_infection_ is the calculated daily infection [10, 11].

## 3. Results

### 3.1. Socio-Demographic Characteristics

A total of 199 households were surveyed, with a total of 379 water samples from various water sources from each household including 15 common dug wells. Characteristics of the respondents from the households are described in Table 1. Almost one-third of the households in this slum area were in the status of rented or shared house, and 16.1% were using shared or common latrine. About 23.1% of the head of family were jobless, and 26.1% had low education status. Tap water was not equally distributed in the whole area; therefore, about 37.7% of the household were likely to use groundwater. Mostly, the drinking water source was from bottled water either refill or branded bottled water while some households keep boiling the raw water sources from ground water or tap water for their drinking water. The river along this area had been acknowledged as giant septic tank since most of the households preferred to dispose the human waste from their latrine directly to the river.

Comparation of monthly expenditure for daily living is shown in Table 2. The average for all monthly expenditures among slum dwellers was USD 279.6 (1 USD = IDR 13,000) with food as the biggest expenditure (29%); however the mean expenditure for tap/drinking water took only a small portion (2.2%) from the total expenditure. This expenditure was used for paying the tap water usage and buying the refill or branded bottled water for daily consumption. Households that still boil the raw water also spend 2.3% of the monthly expenditure for gas fuel intended for cooking and boiling water. Other monthly expenditure such as for smoking, groceries, transportation, and education is bigger compared to the expenditure for tap/drinking water supply.

### 3.2. Quality of Water Sources

**Figure 1:**
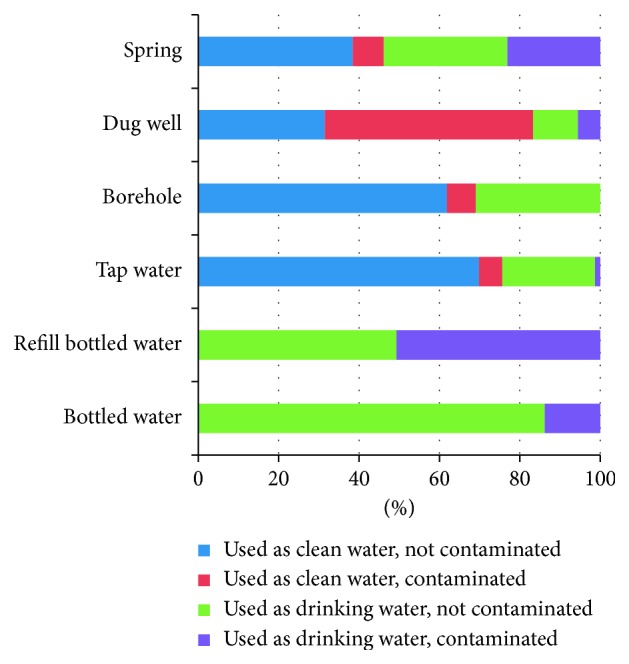
Percentage of contaminated clean and drinking water from various water sources.

Figure 1 illustrates microbiological quality of various types of water sources used by slum dwellers. The type of raw water varies from tap water to groundwater (spring, dug well, and borehole). These types of raw water were used as clean water for daily activities such as cooking, bathing, and washing and also being used as the source of drinking water after boiling. Households that do not use their raw water for drinking would buy bottled/refill bottled water as drinking water source. Percentage of contaminated dug well is higher compared to the other water sources. Furthermore, half of refill bottled water was contaminated, and 3 out of 7 spring water which were used as the source of drinking water found to be contaminated.

Table 3 shows some heavy metal parameters, two different standard solutions ranged from 2 to 50 ppb was used to validate the measured values, which were compared to the certified value. In Table 3, the mean of the heavy metal concentrations was below the Indonesian guideline limit for clean water (water which is used for hygiene and sanitation purposes) and WHO guideline for drinking water except for aluminium in river water. Besides that, the result revealed that there were 10 dug wells and 2 boreholes with concentration of manganese exceeding 500 ppb. Manganese is known as one of the abundant metals in the earth's crust and usually occurs together with iron. Iron and manganese are essential trace elements in human nutrition, and even no health-based guideline value is proposed for iron in drinking water. These metals, however, will affect taste and appearance [8].

### 3.3. Quantitative Microbial Risk Assessment

Using Beta-Poisson model as application of QMRA, the probability of infection can be calculated by incorporating the foreknown values and the average dose of *Coliform* and fecal *Coliform* into Equation (2).  Table 4 suggests the probability of occurrence of fecal *Coliform* infection in all types of water sources tested. Meanwhile, the probability of infection is used to calculate the annual probability using Equation (3) as shown in Table 4. USEPA limits the annual probability value by 10^−4^ per person, yet Haas et al. and Blumental et al. stated that the annual probability can be tolerated until 10^−3^ per person or more, depending on different conditions of the country in which studies are conducted [11, 13]. Currently, the tolerated range of the annual infection probability had not been determined in Indonesia.  Table 4 describes the annual probability of infection due to consumption of dug wells and spring water sources in urban slum areas in the study site that exceeds the tolerated limit.

## 4. Discussion

Since this slum area is located in the city center, tap water connection is available, but only half of the households use tap water as their main water source; other dwellers use ground water sources (borehole or dug-well) and spring. Some of the community still use common dug wells particularly those who stay in rented or shared houses; these types of houses usually do not have their own latrine inside their house, therefore residents use common toilets as the consequences. One common dug-well is usually used by 3–5 houses; however, in certain locations, it can be used by more than 20 houses. One household might use more than one type of water source; sometimes people use tap water and also have dug-well/borehole as a secondary water source.

Interestingly, for drinking water sources, more than half of the communities (66.8%) prefer to choose bottled water either refill or branded bottled water while the others still boil tap or ground water. Refill bottled water is more popular compared to bottled water from branded company among the slum dwellers. Refill bottled water is provided by water refill stations, and the consumers should bring their own water container which is commonly a 19-liter plastic bottle. Some of them buy refill bottled water because of practicality reason and due to the price that is three times cheaper compared to branded bottled water [14]. Monthly expenditure for tap/drinking water is smaller compared to other expenditures; this might be because slum dwellers prefer to boil their raw water or choose refill bottled water.

Due to limitations of capability from the local government company that is responsible for distributing tap water to all parts of the city, tap water is not always distributed 24 hours a day and sometimes only a few days a week and only 2-3 hours at midnight. Lack of sufficient volume of raw water sources, low capability of water treatment production, and a high percentage of leakage in the distribution system contribute to the problem of low-tap water coverage in Bandung city. At the national scale, the water loss due to leakage in piping systems reported ranges between 25% and 30% in metropolitan cities and 30%–35% in smaller cities [15–17]. Therefore, people prefer to use ground water such as dug wells or boreholes as their main water sources. The river basin area is located in the lowest level; consequently, it is quite easy to get water from ground water and spring which are also available in some spots. However, in this slum area, almost all (91%) of the households have no septic tanks and dispose their human and domestic waste directly to the river, and it causes high risk of contamination to their ground water sources.

*Coliform* bacteria existed in all types of water sources, especially in unprotected dug wells and springs, even almost all of unprotected springs are contaminated. *Coliform* contamination may be caused due to contamination originated from latrines without a septic tank surrounding the water source. It is quite interesting that bottled water is popular among slum dwellers; however, we found that half of refill bottled water was contaminated mostly by *Coliform*. This fact is quite important to be considered that the quality of refill bottled water might not be appropriate in the water refill station or recontamination happened in the households seeing as hygiene and sanitation is the substantial problem in the slum area. Previous studies reported that the quality of refill bottled water was not fully supervised, and 25%–40% were still not suitable as drinking water [9, 18, 19]. In this study, only 8 out of 50 bottled water were still contaminated compared to other studies in Iran that found no contamination of *Coliform* in bottled water but still positive for Heterotrophic Plate Counts (HPCs) [20, 21].

Tap water in developed country should be potable and not contaminated by Coliform/E.Coli, however in many developing countries, even the local government treat the tap water before distribute it, ussualy the consumer should boil the tap water before use as drinking water. The previous study from the decentralized municipal desalination plan in Iran showed that even the mean value of physical and chemical parameters complied with the regulation limit, but 10% of HPC samples in the outlet and 14% of samples in the distribution network did not comply with standard [22, 23]. This study showed that half of tap water found to be contaminated in household distribution; this recontamination might be due to some factors such as leakage in the main or household distribution system, low pressure along the pipe distribution, and not fully continuous water flow. Worst sanitation and hygiene condition in the household distribution also contribute to this contamination definitely.

The location of this slum area is in the city center and far away from the industrial area. Therefore, there is no heavy metal concentration found. High concentration of ferrum/iron in ground water sources is due to the geological characteristic of the earth's crust in Bandung city [24]. There is no health-based guideline value for iron in drinking water, however high concentration of iron might cause many esthetical effects due to staining laundry and ceramic as well as high turbidity and colouring the water. Commonly, slum dwellers simply use the conventional filtration method to minimize the iron concentration.

Analysis using QMRA contains three steps: the first is a health-hazard identification followed by an exposure assessment and then a risk characterization. In the hazard identification phase, the catchment-to-consumer system description, the selection of index pathogen, and the selection of hazardous events were elaborated. Although QMRA was previously intended to be applied for a specific pathogen, completing a QMRA for every pathogen that may be transmitted by water would be challenging [25]; thus, microorganism indicators are used instead. In this study, the total *Coliform* and fecal *Coliform* were observed as indicator bacteria in water because nonspecific diarrhea is the most common type of waterborne disease reported in outpatient primary health centers in Indonesia.

The last step of QMRA is the risk characterization, which aims to integrate the exposure information with dose-response analysis to obtain overall risk estimates. High contamination in dug wells and springs in urban slum areas is associated with poor hygiene and sanitation due to the small number of households that use septic tanks. The community is accustomed to disposing human waste directly to the riverbank by using closed/open pipes. Safe excreta disposal is also critical as a first barrier to disease transmission. Therefore, the reduction of morbidity and mortality from infectious diarrheal diseases requires improvements in the quality and availability of water, excreta disposal, and general personal and environmental hygiene. Proper water treatment is extremely important to prevent infection from consuming contaminated drinking water. Since half of refill bottled water did not fulfill the standard, the annual probability of having infection from refilled bottled water as potable water is higher compared to tap water and borehole as raw water. This fact demonstrates that the possibility of recontamination during transportation or inappropriate cleaning methods of the storage among urban slum dwellers is high. The previous study by Sima et al. showed that the usage of water from community-scale water refill stations can reduce the risk of childhood diarrhea [26], but if the quality of refill water is not properly monitored, the community will have risk of consume unstandardized drinking water. Lack of government control on the quality of refill water station production is also an important factor; subsequently, the implementation of an integrated monitoring system for bottled water production is urgently needed.

### 4.1. Limitation of the Study

In this study, the calculation of the annual probability of infection was calculated from raw water; therefore, we cannot compare the risk of potable water between boiling water and bottled water. Heavy metal contamination was tested only from ground water sources and limited only for selected parameters.

## 5. Conclusions

Although the infection probability in bottled water, tap water, and borehole is tolerable, the presence of *Coliform* and fecal *Coliform* in both potable and raw water sources suggest that measures should be undertaken to reduce the risk of infection. The higher probability of infection in dug wells and spring water sources suggests that proper water treatment should be proposed prior to consumption as drinking water. An improvement in the distribution networks of tap water is crucial to reduce the risk of waterborne diseases. The integrated monitoring system to control the quality of refill bottled water production is one of the many essential issues to be prioritized.

## Figures and Tables

**Table 1:** Characteristics of respondents.

Characteristics (*n*=199)	*n*	%
*The house status*		
Owner	145	72.8
Rented	25	12.5
Shared	29	14.7

*Head of family job*		
No job	46	23.1
Civil servant	7	3.5
Business	102	51.2
Employee	44	22.2

*Head of family highest education*		
Elementary	52	26.1
Junior high	27	13.5
Senior high	94	47.7
University	26	12.7

*Type of raw water sources*		
Tap water	103	51.7
Tap water + ground water	14	7.1
Dug-well	48	23.9
Borehole	27	13.8
Unprotected spring	7	3.5

*Type of potable water sources*		
Boiling raw water	66	33.2
Refill bottled water	76	38.2
Branded bottled water	57	28.6

*Distance of ground water sources to the river (n*=110)		
<2 meters	12	10.9
2–10 meters	31	28.2
11–20 meters	11	10
21–50 meters	21	19.1
>50 meters	35	31.8

*Type of human disposal waste*		
Septic tank	7	3.5
Sewerage from city system	4	2
River	182	91.5
Hole in backyard	6	3

*Ownership of the latrine*		
Owner	167	83.9
Sharing with other house	19	9.5
Use of common toilet	13	6.6

**Table 2:** Monthly expenditure in households.

Monthly expenditure (USD) (*n*=199)	Min-max	Mean ± SD	Proportion from total expenditure (%)
Food	5.9–461.5	126 ± 78	29
Smoking	1.4–208	31.8 ± 26	7.3
Rent house (*n*=21)	0.6–192	36.3 ± 42.3	8.4
Health	0.1–169	14.4 ± 25.8	3.3
Education	0.5–538.5	42.8 ± 76	10
Tax/insurance	0.2–269	18.4 ± 47	4.2
Communication	0.4–115	13.4 ± 14	3
Grocery	0.7–154	17.4 ± 21	4
Transportation	0.7–231	23 ± 32	5.3
Services (maid/driver) (*n*=12)	15.4–138.5	60.5 ± 36.5	14
Electricity	1.5–92	18 ± 17.6	4.1
*Tap/drinking water*	**1–58**	**9.7 ± 9.4**	**2.2**
*Gas fuel*	**1.5–61**	**10 ± 9**	**2.3**
Others	0.2–185	4 ± 17	2.9

**Table 3:** Heavy metal concentrations from all types of water sources.

	Concentration in ng/gr (mean ± SD)				
	Al	Mn	Cu	As	Pb
Borehole (*n*=22)	5 ± 5	187 ± 287	4.5 ± 5.2	0.5± 0.2	0.2 ± 0.4
Dug well (*n*=38)	47 ± 79	368 ± 497	3.4 ± 7.6	0.5 ± 0.5	0.2 ± 0.5
Spring (*n*=1)	7	36	1.4	0.1	0
River (*n*=1)	1374	552	4.7	0.8	1.2
Indonesian guideline for clean water	200	500	1000	50	50
WHO guideline for drinking water	200	500	1000	10	10

**Table 4:** The risk of infection due to consumption of various types of water sources.

Type of water source	Concentration of fecal *Coliform* (CFU/100 ml)	Dose of consume fecal *Coliform* (CFU/person/day) (Equation (1))	Probability of infection cause by fecal *Coliform* (Equation (2))	Annual probability (Equation (3))
Bottled water	0.25	2.81	0.58 × 10^−8^	0.21 × 10^−5^
Refill bottled water	2.46	27.09	5.60 × 10^−8^	2.04 × 10^−5^
Tap water	1.35	14.86	3.07 × 10^−8^	1.12 × 10^−5^
Borehole	1.67	18.34	3.79 × 10^−8^	1.38 × 10^−5^
Dug well	1366.67	15033.34	3107 × 10^−8^	1127.9 × 10^−5^
Spring	60	660	136.4 × 10^−8^	49.7 × 10^−5^

## Data Availability

The data used to support the findings of this study are available from the corresponding author upon request.
